# Document or Lose It—On the Importance of Information Management for Genetic Resources Conservation in Genebanks

**DOI:** 10.3390/plants9081050

**Published:** 2020-08-18

**Authors:** Stephan Weise, Ulrike Lohwasser, Markus Oppermann

**Affiliations:** Leibniz Institute of Plant Genetics and Crop Plant Research (IPK) Gatersleben, Corrensstr. 3, 06466 Seeland, Germany; lohwasse@ipk-gatersleben.de (U.L.); opperman@ipk-gatersleben.de (M.O.)

**Keywords:** documentation, genebank, plant genetic resources, agricultural biodiversity

## Abstract

Genebanks play an important role in the long-term conservation of plant genetic resources and are complementary to the conservation of diversity in farmers’ fields and in nature. In this context, documentation plays a critical role. Without well-structured documentation, it is not possible to make statements about the value of a resource, especially with regard to its potential for breeding and research. In particular, comprehensive information management is a prerequisite for the further development of genebank collections. This requires detailed information about the composition of a collection, thus allowing statements about which species and/or regions of origin are under-represented. This task is of strategic importance, especially due to the threats to crop plants and their wild relatives caused by advancing climate change. Both the actual conservation management and the fulfilment of legal obligations depend on information. Hence, documentation units have been established in almost all genebanks worldwide. They all face the challenge that knowledge about genebank accessions must be permanently managed and passed on across generations. International standards such as Multi-Crop Passport Descriptors (MCPD) have been established for the exchange of data between genebanks, and allow the operation of international information systems, such as the World Information and Early Warning System on Plant Genetic Resources for Food and Agriculture (WIEWS), the European Search Catalogue for Plant Genetic Resources (EURISCO) or Genesys.

## 1. Introduction

For many centuries, humans have been taking advantage of the plant world and adapting it to their needs. The resulting diversity of useful plants is the main source of food for humans and animals [1]. In addition to the nutritional aspect, plants also supply raw materials for the chemical and pharmaceutical industries and are renewable energy sources [2,3]. Global biodiversity is severely threatened by human intervention, not the least against the background of advancing climate change [4]. This also applies to the diversity of crops.

Genebanks play an important role in the long-term conservation of these plant genetic resources [5]. They complement the conservation of diversity in farmers’ fields and in nature. There are about 1800 genebanks worldwide, more than 600 of them in Europe [6]. The important genetic diversity stored in genebanks can provide new impulses for research and breeding, e.g., by introducing new alleles into existing breeding stocks [7], which have a morphologically and physiologically beneficial effect on the characteristics of the plants. Besides maintenance and regeneration, an important task of genebanks is therefore the phenotypic characterisation of accessions [8].

In this context, documentation plays a crucial role [9,10]. Imagine a supermarket with shelves where all the goods are unlabelled. In addition, only a few persons know what is on which shelf. There are no records of where the goods come from, how old they are and for how much they have to be priced. It is obvious that such a store cannot work. The same holds true for genebank holdings. Without having as much information as possible available in a well-structured way, it is not possible to make informed statements about the value of a resource, especially with regard to its breeding and research potential. The wealth of information covers many areas, from data necessary to optimally manage collections over genebank basic data (passport data) to phenotypic and comprehensive genetic data. One of the greatest challenges of genebanks, apart from the conservation of accessions, is the management of these data [11,12].

This article aims to provide an overview of the relevant systems and structures of plant genetic resources documentation in genebanks. The development of documentation, the current situation and international cooperation as well as the specification of data for long-term and sustainable conservation management are described. In addition, the current needs for integrative information management based on several requirements for future genebank documentation are described.

## 2. Information Required

For the long-term conservation and use of plant genetic resources, it is necessary to document a large amount of information at various levels, especially for the identification and characterisation of accessions.

### 2.1. Basic Data

Basic information on plant genetic resources is contained in the passport data. They serve in particular to identify the material and contain information such as the accession number, the scientific name and information on the origin and acquisition of the material (year of acquisition, donor, collecting mission, location of collecting). Ideally, these data are following the standard of the Multi-Crop Passport Descriptors (MCPD) [13,14], which has been developed as a uniform, global format. This corresponds with the recommendations of the Genebank Standards [15] of the Food and Agriculture Organization of the United Nations (FAO), which were developed by the FAO Commission on Genetic Resources for Food and Agriculture (http://www.fao.org/cgrfa/).

The geographical origin of a genebank accession (particularly in connection with environmental data) can provide information on possible adaptations to biotic/abiotic stress factors. Such data should be complemented by information on the type of material (biostatus, e.g., wild form, landrace, etc.). Other important data include phenotypic characterisation of the individual accession, including morphological and agronomical traits.

This basic information helps to identify the individual samples of the genetic resource and to estimate its value, especially its potential for breeding, but also for research.

### 2.2. Stable and Unique Identifiers

Genebanks have been existing for many decades. This long period of time implies that the conditions at genebanks have been and continue to change. On the one hand, changes result from technological progress. This means that both the type of storage and the data itself must be adapted. On the other hand, social, political and economic changes occur, which lead to organisational changes in genebanks. This means that the description and use of plant genetic resources are also subject to constant change and may result in different identifiers for an accession over the time. In addition, the exchange between genebanks is common in order to establish conservation security through targeted safety duplication and to complement collections in the individual countries. Moreover, material is supplied to researchers and breeders, but also to other users.

Before the introduction of information systems spanning several collections, a local, sometimes temporary, unambiguous identification of an accession was sufficient. However, changing identifiers lead to chains of identifiers of an accession and make it more difficult to trace transferred material. Even within a collection, unambiguity could not always be guaranteed, for example, in the case of multiple accession numbers for the same material in the course of time. Identical identifiers in aggregating information systems also pose a problem [16]. This led directly to the consideration of introducing a system that would provide unique and stable identifiers for genebank accessions [17]. Of the various approaches that can be considered for this purpose, digital object identifiers (DOIs) appear to be the most common. The idea of the DOI dates back to the 1990s [18]. A DOI is a unique and permanent digital identifier of a (digital) object. For the description of the object, metadata are associated with it. The name resolution of a DOI is performed using a handle system such as doi.org. The DOI system was introduced in 2000 and is maintained by the International DOI Foundation (IDF) [19]. The core is a central database. It contains the URL under which the referenced object is currently available. The organisation that has registered the DOI is responsible for updating the database entry whenever the metadata are changed. Not the least due to the support by the Secretariat of the International Treaty on Plant Genetic Resources for Food and Agriculture (ITPGRFA), DOIs have established themselves as a quasi-standard for plant genetic resources material [20]. An undeniable advantage of DOIs is their high acceptance in the scientific community. However, it should be critically noted that accessions of plant genetic resources are not non-modifiable digital objects. Data describing accessions are generally subject to changes and additions.

Moreover, it is an indispensable task of the documentation units of the genebanks to map the historically used identifiers of the accessions to these new, unique identifiers.

### 2.3. Data for Collection Development

What is needed to recognise which parts of a collection are over- or under-represented? First and foremost, the composition of a collection naturally depends on its exact purpose. In addition, as comprehensive information as possible on individual accessions is a further essential prerequisite for the further development of genebank collections. With the help of the botanical determinations of accessions, a genebank collection can be examined to determine the extent to which the gene pool of a genus is completely represented or which species/subspecies are missing. In addition, the within species/subspecies diversity should be adequately represented. By adding geographical information, it is possible to specify more precisely from which regions material is missing and should be collected. Such detailed information on the composition of a collection is necessary to perform gap analyses [21,22,23]. In addition, precise geographical data can be used to draw conclusions about ecological conditions. This task is of strategic importance, especially because of the threat posed to crops and their wild relatives by progressing climate change [24]. 

The comparison of data on accessions also allows statements to be made about potential duplicates within a collection. Nevertheless, this task is not trivial. A reliable statement can only be made by the joint analysis of phenotypic, genotypic and passport data in combination with comparative cultivations [25,26].

By analysing combined data from different genebanks, it is also possible to check which accessions are maintained in other collections and, if necessary, could be obtained from there [27,28]. Such comparisons are an important means of expanding a collection in a targeted manner through exchange with other genebanks. This can also contribute to making the genebank work more effectively. One approach that pursues this goal is the European Cooperative Programme for Plant Genetic Resources (ECPGR) initiative, A European Genebank Integrated System (AEGIS, https://www.ecpgr.cgiar.org/aegis/) [6,29]. AEGIS aims to identify which accession in different genebanks is the most appropriate accession (MAA). In this case, genebanks participating in AEGIS undertake to assume responsibility for conservation and to maintain this accession according to uniform standards in the long term.

### 2.4. Securing the Legal Status of Acquisitions

There are international agreements governing the conservation and sustainable use of plant genetic resources as well as access and benefit sharing (ABS), in particular, the Convention on Biological Diversity (CBD, https://www.cbd.int/), which entered into force in 1993, and its supplementary agreement, the Nagoya Protocol of 2014 (Nagoya Protocol on Access to Genetic Resources and the Fair and Equitable Sharing of Benefits Arising from Their Utilization to the Convention on Biological Diversity, https://www.cbd.int/abs/about/). To this end, it is essential to document the origin of genebank accessions, as well as the time of inclusion in a collection and existing collecting permits.

In accordance with the CBD, the International Treaty on Plant Genetic Resources for Food and Agriculture (ITPGRFA, http://www.fao.org/plant-treaty/) came into force in 2004 and the signatory states committed themselves to conserve, characterise and evaluate plant genetic resources and to ensure their sustainable use. The main component of the Treaty is the Multilateral System of Access and Benefit Sharing (MLS), which regulates facilitated access to plant genetic resources that have been included in the MLS and the equitable sharing of the resulting benefits. A Standard Material Transfer Agreement (SMTA, http://www.fao.org/plant-treaty/areas-of-work/the-multilateral-system/the-smta/en/) was created for this purpose. The reporting obligations attached to the SMTA are usually part of the tasks of the documentation units of the genebanks.

### 2.5. Material Management Data

The actual conservation management in the genebanks is also dependent on information, e.g., on storage conditions and location, and the quality and quantity of seeds and other plant propagules at accession level, in order to enable efficient and effective genebank management. This includes information such as germinability or storage quantity as well as information on health tests that have been carried out or are pending. In addition, information on the regeneration of the individual accessions must be documented as well as how and where the material is stored (e.g., active or base collection, cold store, shelf, safety duplication sites) [15]. Depending on size and information technology equipment, the solutions of the individual genebanks differ [12,30].

### 2.6. Data to Protect Against Losses

How is it generally attempted to protect plant genetic resources in genebanks from loss? A fundamental idea of genebanks is the protection against losses of accessions or entire collections, e.g., in case of war or catastrophe. The FAO Genebank Standards [15] recommend to realise this by intentional duplication of unique accessions in different genebanks of geographically distant areas. In recent years, the Global Seed Vault (https://www.croptrust.org/our-work/svalbard-global-seed-vault/) in Svalbard was introduced as an additional safety backup system [31,32,33]. That this has its justification is evident in the example of the genebank of the International Center for Agricultural Research in the Dry Areas (ICARDA), which was reconstructed from security samples in Morocco and Lebanon (https://www.seedvault.no/news/withdrawal-of-icarda-aleppo-seeds-accomplished/). However, such a mechanism has not yet been established for the associated data. It also does not make sense to save data as a black box, as is possible with the physical material. In the case of loss of material, information about where safety duplicates are located, but also who did receive this material (both donors and recipients of material samples), are the keys to finding the material again. Unfortunately, this information is primarily in the genebank, which could be lost. Usually only the basic passport data are shared along with safety duplicates, in some cases not even this. In addition, not all genebanks have a strategy and the ability to secure data which goes beyond this. Similar to the idea of the Global Seed Vault, a global data safe or corresponding, connected repositories would be one way to meet this challenge. Of course, this must not result in losing reference to the physical material. The International Nucleotide Sequence Database Collaboration (INSDC, http://www.insdc.org/) shows that such an approach can work [34,35]. The INSDC dates back to the 1980s and ensures the continuous synchronisation of DNA and RNA sequence data from the three major international systems, GenBank (https://www.ncbi.nlm.nih.gov/Genbank/), the DNA Data Bank of Japan (DDBJ, https://www.ddbj.nig.ac.jp/) and the European Nucleotide Archive (ENA, https://www.ebi.ac.uk/ena).

## 3. Information Management for Genetic Resources Conservation

### 3.1. Documentation Development

The main intention of genebanks is to conserve collections of plant genetic resources for posterity. This means that the documentation of the material must also be ensured across generations. In order to meet the resulting requirements, documentation units have been established in almost all genebanks worldwide. They all face the challenge that the knowledge about the genebank material must be continuously managed, supplemented and passed on across technical and personnel generations. The knowledge must not be tied to individual persons alone. Some genebanks are many decades old, which means that there have been several changes of personnel during this period and possibly also one or more predecessor institutions. Ideally, however, there should still be complete documentation of all accessions of plant genetic resources acquired, maintained and distributed, covering the entire period since the foundation of the genebank.

Historically, the documentation structures and methods of the management of genebanks in the first decades were influenced by experiences from botanical gardens and focused on breeding activities. Accordingly, there was a collection-oriented documentation of the origins, often in the form of collecting mission reports, and a practice-oriented documentation of the collections as inventory books and cultivation documentation. The taxonomic classification of the accessions was an important ordering criterion. Many genebanks regularly created a catalogue, a so-called Index Seminum, to make their holdings publicly accessible. Depending on the size and focus of the genebank, more or less complex index card systems and registries were established. Archives with correspondence and collecting mission reports supplemented these, but often only with weak linkage to collection management. These document holdings were difficult to search and exploit. Due to their limited storage capacity, the first electronic systems were therefore established parallel to paper documentation in order to facilitate searchability, filtering and presentation of the information. With the advancing technical development of databases and spreadsheet programs, different IT solutions were created depending on size, focus and organisational form (centralised or decentralised) of the genebank, which increasingly replaced paper documentation [36,37,38,39,40].

Many larger genebanks have implemented in-house genebank information systems in recent years, e.g., GENIS [41], GBIS [42] or GBIMS [43]. In addition, there was also cooperation between genebanks, sometimes across national borders. For example, the management system SESTO (https://sesto.nordgen.org/) was developed for the joint use of the Nordic countries and was operated by the Nordic Genetic Resource Centre (NordGen) in Alnarp, Sweden. SESTO was also used by the genebanks of the Baltic countries for documentation purposes. However, even today there are still a large number of smaller collections that do not have these capabilities and manage data with simpler means, such as MS Excel lists [44]. For those, the freely available system, GRIN-Global (https://www.grin-global.org/) [45], is increasingly establishing itself as an alternative to proprietary systems, e.g., as seen in Barata et al. [30]. This system was originally developed for the Germplasm Resource Information Network (GRIN) of the United States Department of Agriculture (USDA) and has been made available as an open source version jointly by the Global Crop Diversity Trust, Bioversity International and the USDA’s Agricultural Research Service since 2011. GRIN-Global enables to manage phenotypic data in addition to the basic passport data. Furthermore, the system allows the maintenance of material management data, e.g., germination rates or existing storage quantities. The data can be curated via a corresponding interface. Furthermore, GRIN-Global has an online search and ordering system. This represents a major step forward for structured and sustainable documentation. The Genebank Information System (GBIS) [42] of the German Federal ex situ Genebank for Agricultural and Horticultural Crop Species is following a similar line. Just like GRIN-Global, GBIS allows to manage different types of data (passport and phenotypic data, management data, plant health tests, germination rates, orders, etc.). An online search and ordering system is also publicly available. However, in contrast to GRIN-Global, GBIS is explicitly designed for use in a single genebank. For this purpose, the system is fully integrated into the specific work processes. In this context, GRIN-Global and GBIS shall serve as examples of two different philosophies in the development of information systems in genebanks.

### 3.2. Current Situation

In order to provide an overview of the current situation of information management in genebanks, the authors conducted an ad hoc survey among the National Inventory Focal Points of the European Search Catalogue for Plant Genetic Resources (EURISCO) network (see below). They were asked whether they could provide information on (1) how the genebanks in their respective countries manage their data and (2) which data are managed in addition to basic passport data. From 40 persons contacted, 30 replied. Even though this survey is not representative and certainly reflects only a part of the overall situation, it provides a basic overview. A more extensive survey, aiming in particular at perspective approaches to the future development of the documentation systems in the individual countries, would be a logical consequence, but was not feasible in the context of this article.

**Figure 1:**
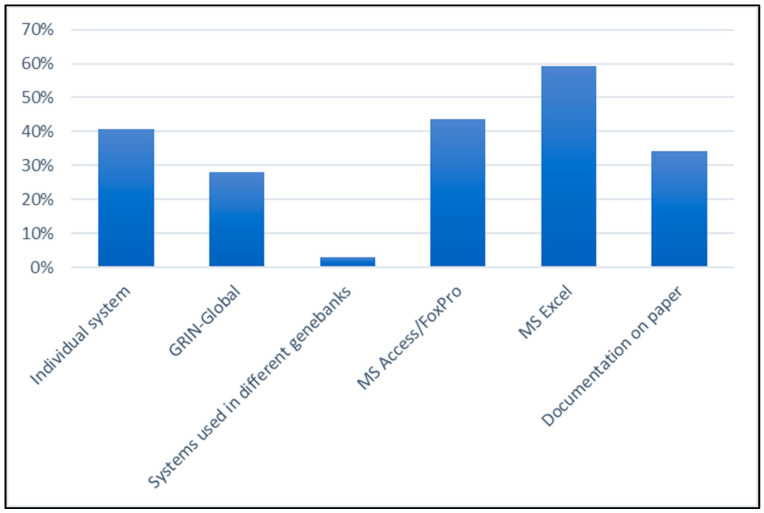
Overview of the management systems used in various genebanks. Different systems are often used depending on the type of data. Therefore, multiple answers were possible.

As expected, the information management in genebanks is very diverse. Since the data of different domains are often managed in different systems, there were multiple answers. Forty percent of the respondents stated that individual information systems are used in the genebanks. Beginning in July 2020, the Nordic and Baltic countries started to use a joint system based on GRIN-Global (Nordic Baltic Genebanks Information System (NBIS), https://www.nordic-baltic-genebanks.org/gringlobal/). Thus, 28% in total use the GRIN-Global system, while only 3% apply other systems that are used in different genebanks. More than 40% use MS Access or FoxPro for data management. The most widespread use is MS Excel (59%). Thirty-four percent still document in paper form (Figure 1).

All respondents indicated that they manage passport data. This was to be expected because this is the basic information for genebanks. In addition, it was stated that more than 90% also hold phenotypic data and information on seed stocks. Sixty-nine percent also manage data on seed orders.

### 3.3. International Collaboration

Despite ever better IT support through genebank information systems, these remained largely isolated from each other. Since the 1980s, a start has been made on compiling data on accessions of one or more crop species obtained in genebanks in a region or even worldwide into databases, the so-called Central Crop Databases (CCDBs) [46]. Two of the earliest Central Crop Databases are the European Barley Database [47] and the European Prunus Database [48]. The CCDBs have strengthened the cooperation between genebanks and have been made possible through networking genebanks and collections. In addition, the CCDBs aimed to make genebank material more accessible to users and to identify possible duplicates between the individual collections. However, these goals could only be achieved to a limited extent, particularly due to the limited availability of these databases and their low data quality or lack of such data [49]. Another major challenge was the long lack of uniform standards for the description and exchange of passport data. In 1997, a first draft of the Multi-Crop Passport Descriptors (MCPD) was presented [50], which was subsequently developed into a globally accepted standard [13,14].

Aggregating platforms and databases for a cross-genebank search for suitable accessions have also been and are being developed. MCPD and Darwin Core [51,52] have been established for the exchange of passport data between genebanks and these platforms. It is only through such standards that it is possible to feed and operate international information systems such as the World Information and Early Warning System on Plant Genetic Resources for Food and Agriculture (WIEWS, http://www.fao.org/wiews/), EURISCO (http://eurisco.ecpgr.org/), Genesys (https://www.genesys-pgr.org/) or the Global Biodiversity Information Facility (GBIF, https://www.gbif.org/), which combine the information as homogeneously as possible and make it available beyond the boundaries of the individual genebank collection.

While the intention of WIEWS is to provide periodic, country-driven assessments of the plant genetic resources conservation status for FAO, the European Search Catalogue for Plant Genetic Resources (EURISCO) provides detailed accession-specific information on the majority of European collections [53]. Of the approximately 600 European collections of plant genetic resources, more than 400 provide their accession-level data to EURISCO. Information on the geographical location of the genebanks can be found on the EURISCO website. The development of EURISCO started in 1999. For this purpose, a network of national inventories covering 43 countries was successively established. These national inventories bring together the data from the respective collections of their countries and then make them available to the EURISCO information system in a coordinated manner. EURISCO currently documents more than two million genebank accessions, the data of which are regularly updated. The MCPD standard plays a key role here.

Another approach is the Genesys information system, which has been developed since 2008 and is based on the System-wide Information Network for Genetic Resources (SINGER) system. SINGER was an integrated system for the management and exchange of data on plant genetic resources held in Consultative Group on International Agricultural Research (CGIAR) genebanks (https://cgiar.org/). This system was originally developed in 1994 and was put on a new technological basis with Genesys. In addition, EURISCO as the European hub and the US GRIN system as the North American hub regularly feed their data into Genesys as well. A source of additional Australian data is the University of Queensland’s Crop Trait Mining Informatics Platform [54]. The aim of the platform is to make information available through the existing Genesys system to support the development and use of plant genetic resources. Both EURISCO and Genesys comprise passport data as well as phenotypic data.

The above-mentioned GBIF is both an international network and an infrastructure designed to make data on global biodiversity freely and permanently available. For this purpose, standards and tools for the exchange of information are provided. With its all-encompassing approach to aggregating data from all areas of biodiversity, GBIF holds a special position. In the plant sector, this network mainly includes data from natural history collections, such as botanical gardens, herbaria and other biodiversity databases, but also includes data on plant genetic resources, which are made available either via aggregators, such as EURISCO, or directly through the genebanks.

The systems just described have made a significant contribution to standardising the documentation of plant genetic resources and improving international cooperation. They make it easier to find information on accessions maintained in a large number of genebanks. In addition, they provide positive impulses for the coordination of the conservation of collections. In particular, the European approach AEGIS is being promoted as a virtual European genebank and aims to define the efficient conservation of genebank material using uniform standards.

In general, networks at the regional and/or international level play an outstanding role in developing the above approaches. They contribute to bundling existing strengths and resources in order to master common challenges. In this context, the European Cooperative Programme for Plant Genetic Resources (ECPGR, https://www.ecpgr.cgiar.org/) should be mentioned, under which a large number of Central Crop Databases as well as the EURISCO system were developed. From the field of natural science collections, the Distributed System of Scientific Collections (DiSSCo, https://www.dissco.eu/) approach should be mentioned here. DiSSCo aims to curate and make accessible the holdings of European natural science collections according to uniform criteria. This approach could provide additional impulses to advance the international networking of genebanks and their documentation.

In the context of international cooperation, reference should be made here once again to the International Treaty on Plant Genetic Resources for Food and Agriculture (ITPGRFA, see above). Article 17 of the Treaty contains the vision for the development of a global information system. Since 2015, a Global Information System (GLIS) for plant genetic resources on the basis of existing information systems has been underway. The goal of the GLIS is to provide a global entry point for knowledge and information to support the conservation, management and use of plant genetic resources (http://www.fao.org/plant-treaty/areas-of-work/global-information-system/). Besides the GLIS DOI portal (https://ssl.fao.org/glis/) of the Treaty Secretariat, WIEWS, Genesys, EURISCO and GRIN-Global are major components of GLIS.

Successful international cooperation, also in the field of documentation, supports the fulfilment of the Sustainable Development Goals (SDG, http://www.fao.org/sustainable-development-goals/) developed by 193 member states of the United Nations.

Finally, it is worth mentioning the international DivSeek network (https://divseekintl.org/), which is a worldwide collaboration to support the creation, integration and exchange of data on plant genetic resources. A number of working groups have been established for this purpose.

## 4. Challenges

As already mentioned, genebanks manage more than just passport data and material management data on accessions. For plant genetic resources, however, there are no generally accepted standards for capturing and exchanging data other than passport data [55]. This is a particular challenge for phenotypic and genotypic data. 

Phenotypic data are collected in genebanks for various reasons. On the one hand, they provide important information for a better exploitation of the collection material. On the other hand, they support the management of collections, for example, they serve to ensure the quality of seed multiplication. Many genebanks collect a range of phenotypic and agronomic traits during each multiplication cycle to characterise the material, but also to detect potential mixing or swapping of material. This is particularly important for cross-pollinated species. Initiatives to harmonise the collection of phenotypic data in the field of genebanks have been underway since the late 1970s. The IPGRI/Bioversity descriptor lists developed for different crop species are a good example of this, e.g., [56,57,58]. However, it never was possible to achieve general acceptance. In many genebanks, the phenotyping of the material is based on such lists, but they have often been further developed and adapted to the respective practice. All data collected in this way are of limited comparability. In addition, there is a large number of scientific experiments, not carried out by the genebanks themselves, in which phenotypic data are collected from genebank material. The European information system EURISCO (see above) also collects phenotypic data [53]. Due to the problems just described, it was decided here not to standardise the data itself, but only the exchange format. This is a minimum consensus format that only contains the fields that are absolutely necessary. The idea behind this was to first gather a critical mass of data from which it is worthwhile to start a discussion with providers and users about harmonising traits and methods [59]. In this context, there are current approaches that aim to improve the comparability and traceability of phenotypic data, e.g., by recommendations for more extensive documentation of metadata such as Minimum Information About a Plant Phenotyping Experiment (MIAPPE) [55,60,61]. Mapping of traits and methods to ontology terms, such as Crop Ontology [62,63], is also promising.

Platforms for combining and analysing the data from the different domains are also essential, and can be adapted to the needs of projects or communities. A promising and already successfully used approach is the platform Germinate, developed by the James Hutton Institute, which is used in various projects to represent their data [64].

In addition to phenotypic data on genebank collections, genotypic data are increasingly coming into focus. Genotypic data can help to better exploit the treasures stored in genebanks [65,66]. Against this background, reference should be made here to pilot projects, which aim to exploit entire collections on a molecular level. For example, the BRIDGE project carried out the genotyping of the entire barley collection with more than 20,000 accessions of the German Federal ex situ Genebank for Agricultural and Horticultural Crop Species [67]. The analysis of such genome-wide genotyping-by-sequencing data provides the basis for gene annotation, marker-assisted selection or a better understanding of the population structures of globally domesticated crops, to name a few. In addition, molecular data can also be used to improve the curation of genebank collections, for example, with regard to potential duplication [68].

Moreover, such approaches can contribute significantly to the long-term development of traditional genebanks into biodigital resource centres. This means integrated centres which, in addition to the actual plant genetic resources, also provide a large amount of associated information from various data domains and thus enable better and more targeted access to the material [26,69]. According to this objective and based on first experiences, e.g., from the above-mentioned BRIDGE project, a number of further research projects with international participation are currently taking place. Examples are the Activated GEnebank NeTwork (AGENT, https://www.agent-project.eu/) and Intelligent Collections of Food Legumes Genetic Resources for European Agrofood Systems (INCREASE, https://increase-h2020.eu/) projects.

However, new challenges arise from this thoroughly gratifying development. Single-seed descent (SSD) lines must be generated for genotyping. However, depending on the type of accession, these SSD lines reflect only a selection from the original accessions. This is particularly problematic in the case of populations. Strictly speaking, the SSD lines would have to be preserved as precision collections in addition to the original accessions. The challenge for the documentation is that the data do not explicitly represent the SSD lines either. For example, it is doubtful that an SSD line that is derived from a landrace can still be called a landrace, since the character of a landrace is greatly lost through the selection of a single-seed descent. Conversely, the data obtained by genotyping an SSD line cannot be transferred to the entire accession. The same applies to phenotypic data that is not necessarily valid for both the original accession and the SSD line created from it. If, for capacity reasons, not all SSD lines can be preserved, assigning the data collected to the original accessions without comment should be avoided.

## 5. Conclusions

In this article, an overview was given of the most important topics, systems and structures of the documentation of plant genetic resources in genebanks. Furthermore, existing challenges were described.

In the context of the conservation of plant genetic resources, structured documentation of the material is essential. This includes a variety of basic information, such as origin or legal status, but also data on conservation management, such as storage quantities, germinability, etc. In addition, phenotypic and agronomic characterisation play an important role. Only through this it is possible to exploit the potential of the conserved material for research and breeding.

Over the past decades, there have been significant efforts to intensify cooperation between genebanks in order to conserve plant biodiversity in the best possible way. To this end, networks have been established, such as the European Cooperative Programme for Plant Genetic Resources. Such networks have been an important basis for the development of international aggregator systems such as EURISCO or Genesys, which serve as central entry points for the search for plant genetic resources.

These information systems are currently limited to passport data and phenotypic data. In terms of a sustainable use of genebank collections, it seems promising to continuously expand the interactions between the globally available information systems and to enrich the existing information on genebank accessions with additional data from other domains. In this context, a number of pilot projects have been successfully carried out in the recent past, which aimed at the molecular exploitation of genebank collections. These promising approaches must be continued in the future.

In order to meet the requirements of the modern research landscape, which is characterised by constant diversification and big data, the information systems of the genebanks must be able to connect with other data sources and to make their data available in a network. The linkage with each other and with other data domains leads to new knowledge about plant genetic resources, which ultimately improves their usability and the reputation of the genebanks.
